# Beneficial Effect of Multidomain Cognitive Training on the Neuropsychological Performance of Patients with Early-Stage Alzheimer's Disease

**DOI:** 10.1155/2018/2845176

**Published:** 2018-07-11

**Authors:** Anastasia Nousia, Vasileios Siokas, Eleni Aretouli, Lambros Messinis, Athina-Maria Aloizou, Maria Martzoukou, Maria Karala, Charalampos Koumpoulis, Grigorios Nasios, Efthimios Dardiotis

**Affiliations:** ^1^Department of Speech and Language Therapy, Higher Educational Institute of Epirus, Ioannina, Greece; ^2^Department of Neurology, University Hospital of Larissa, University of Thessaly, Larissa, Greece; ^3^Lab of Cognitive Neuroscience, School of Psychology, Aristotle University of Thessaloniki, Thessaloniki, Greece; ^4^Neuropsychology Section, Department of Neurology, University of Patras Medical School, Patras, Greece; ^5^Olympion Rehabilitation Hospital, Ioannina, Greece

## Abstract

**Background and Purpose:**

There is an increasing interest in the effect of nonpharmacological interventions on the course of patients with Alzheimer's disease (AD). The objective of the present study is to determine the benefits of a structured, multidomain, mostly computer-based, cognitive training (MCT) οn the cognitive performance of patients with early-stage AD.

**Method:**

Fifty patients with early-stage AD participated in the study. Patients were randomly allocated either to the training program group (*n* = 25) or to a wait list control group (*n* = 25). The training program group received computer-assisted MCT and linguistic exercises utilizing pen and paper supplemented by cognitive-linguistic exercises for homework. The duration of the MCT intervention program was 15 weeks, and it was administered twice a week. Each session lasted for approximately one hour. Objective measures of episodic memory, delayed memory, word recognition, attention, executive function, processing speed, semantic fluency, and naming were assessed at baseline and after the completion of the program in both groups.

**Results:**

Analysis showed that in controls, delayed memory and executive function had deteriorated over the observation period of 15 weeks, while the training group improved their performance in word recognition, Boston Naming Test (BNT), semantic fluency (SF), clock-drawing test (CDT), digit span forward (DSF), digit span backward (DSB), trail-making test A (TMT A), and trail-making test B (TMT B). Comparison between the training group and the controls showed that MCT had a significant beneficial effect in delayed memory, naming, semantic fluency, visuospatial ability, executive functions, attention, and processing speed.

**Conclusions:**

The study provides evidence of a beneficial effect of MCT with an emphasis on cognitive-language performance of patients with early-stage AD. Considering the limited efficacy of current pharmacological therapies in AD, concurrent computer-based MCT may represent an additional enhancing treatment option in early-stage AD patients.

## 1. Introduction

Alzheimer's disease (AD) is a slowly progressive neurodegenerative disorder, affecting memory, executive function, visuospatial skills, and language [1–4]. Depending on the stage of the disease, the deficits differ. In comparison to those with mild cognitive impairment (MCI), individuals with early-stage dementia appear to perform more poorly in more than one cognitive domain, leading to a more substantial interference in daily activities and independent function [5], which may negatively affect the quality of life of patients and their caregivers [6–8]. Unfortunately, current pharmacological therapies have limited efficacy in reversing or even halting AD progression [9, 10].

A number of nonpharmacological approaches, aiming at brain neuroplasticity, such as cognitive training [11, 12], cognitive stimulation [13], and cognitive rehabilitation [1], have entered the picture as potential strategies for the prevention and treatment of the cognitive and behavioral symptoms of AD [7, 14]. Emerging rehabilitation approaches for people with AD encompass a variety of techniques (e.g., task-oriented training, strategy training, and individualized training).

According to systematic literature review [15], cognitive training is more effective when compared to cognitive stimulation and cognitive rehabilitation in patients with MCI and early-stage AD. Cognitive training is a guided set of standard tasks that replicate specific cognitive functions. Each task has several levels of difficulty, tailored to the individual's ability, and it is offered in individual sessions. On the contrary, cognitive rehabilitation aims to improve everyday functions by using compensatory individual approaches. Lastly, cognitive stimulation aims to improve cognitive function, not adaptive tasks, through significant use of orientation or reminiscence therapy, offered in group sessions while placing emphasis on social interaction [1, 11, 15, 16].

Limited evidences of positive effects of various cognitive-improving techniques on cognitive functions, however, have been reported [15, 17]. Moreover, several approaches of cognitive training have been developed so far: some focus on a specific cognitive domain, for example, memory or executive functions [16, 18, 19], others on two or three cognitive domains simultaneously [20–22], while others focus on behavioral and psychological symptoms of AD [1, 23]. Furthermore, Bahar-Fuchs et al. noticed that, although many studies claim that language training was included in their cognitive-training program, none of them provided specific information about the domains of the language which were trained as well as about the content of the language tasks [24]. Therefore, it seems that a multidomain cognitive training with an emphasis on language could be the most appropriate and effective approach.

Another issue under consideration, though, is that most of the cognitive-training programs depend on the therapist and many sessions are required. As such, they might not be ideal when it comes to patient comfort. For this reason, more recent studies suggest the use of computer training as opposed to traditional training with the use of paper and pencil [25]. In particular, paper and pencil cognitive training has been reported to be more effective when it is ecologically designed [26] and administered in groups [27] than computer-based programs. A recent study of Tsolaki et al. in patients with MCI reported that the pen and paper cognitive training had better results in general cognitive function, learning ability, delayed verbal recall, visual memory, verbal fluency, and visual selective attention compared to computer-based cognitive training [28].

On the other hand, Man et al. investigated the effectiveness of a computer-based memory-training program versus the same program administered by paper and pencil, in people with questionable dementia [29]. The results demonstrated that, although both programs improved participants' memory performance, the computer memory training improved more cognitive abilities of participants than the paper and pencil training. Shao et al. reported that computer cognitive training has certain advantages for patients with early-stage AD [25]. First of all, it is an effective and convenient method. Moreover, it offers self-paced, individualized training, which sets the initial level of task difficulty according to the baseline competency of participants and gradually adjusts it as their performance improves.

Taking the aforementioned into consideration, in the present study, we used a multidomain cognitive training (MCT) with an emphasis on language. We believe that such a training has many advantages: (a) it focuses on both cognitive functions and language skills; (b) it is user-friendly to patients with AD, as the computer is connected to a special input panel using the commercially available RehaCom software package; (c) the cognitive performance of the patient can be assessed even after each session; (d) the level of difficulty of the tasks does not depend on the therapist; (e) it adapts to the needs of each patient; and (f) it provides an objective measurement of the patient's performance, limiting the possibility of a performance under/overestimation.

In particular, in the present study, we were interested in the beneficial influence of MCT on the neuropsychological performance of patients with early-stage AD, by using a computerized program that would focus on several cognitive domains simultaneously, accompanied by language exercises with pen and paper, which concentrate mainly on language deficits. The study is based on the primary hypothesis that patients who are receiving the 15-week computer-assisted cognitive-linguistic training intervention with an emphasis on alleviation of episodic memory, information processing speed, executive functions, attention, confrontation naming, semantic fluency, and syntax would show improved performance on standardized neuropsychological and linguistic measures compared to the control group which will attend the standard clinical care. Our secondary hypothesis was that the beneficial effects would translate to improved activities of daily living and functional communication.

## 2. Method

### 2.1. Participants

Participants were included in the study if they fulfilled the following criteria: (1) a diagnosis of AD according to the National Institute of Neurological and Communicative Disorders and Stroke and the Alzheimer's and Related Disorders Association (NINCDS-ADRDA), (2) mild (early-stage) AD (Clinical Dementia Rating score CDR = 1 [30] and Montreal Cognitive Assessment (MoCA) score of 16/30 or higher [31]), (3) age between 60 and 80 years, and (4) at least 6 years of education. Exclusion criteria were (1) presence of major psychiatric disorders (e.g., psychotic symptoms or disorders, alcohol or illegal drug abuse, and depression), (2) presence of another neurological disorder (e.g., stroke, epilepsy, and traumatic brain injury), and (3) visual/hearing impairment or writing/reading disability sufficient to impair the performance in the assessment and the training. All participants had undergone clinical neurological assessment, blood tests, and brain magnetic resonance imaging scans that presented no evidence of other diseases.

### 2.2. Procedure

#### 2.2.1. Neurological, Neuropsychological, and Language Evaluation

In order to assess the participants' cognitive status (attention, processing speed, executive function, delayed and episodic memory, and recognition) and language abilities (naming, semantic fluency, and word recall), the following were used: (a) Montreal Cognitive Assessment (MoCA; [32]); (b) trail-making test, part A and part B (TMT A and TMT B; [33]); (c) digit forward and backward tests [34]; (d) repeat and word recognition and delayed memory; (e) verbal fluency [35]; and (f) Boston Naming Test (BNT; [36]). In addition, participants were assessed for depressive symptomatology by using the Geriatric Depression Scale (GDS) [37], whereas possible impairment of their everyday activities was estimated via the Instrumental Activities of Daily Living (IADL) questionnaire [38]. The participants' evaluation was performed by both a neurologist and an experienced neuropsychologist before and after the intervention program.

#### 2.2.2. Multidomain Cognitive Training (MCT) with an Emphasis on Language Abilities

Patients were randomly assigned [39] to either attend the MCT program or be placed in the control group and receive the usual standard clinical care. Concerning the initial assessment stages, the neurologist was not aware of the neuropsychological evaluation, and likewise, the neuropsychologist was not aware of the neurologic assessment. Therefore, their individual results did not affect the diagnosis process and the selection, consequently, of the patients for both groups. An individual therapist finally assorted the patients in the groups (training and control). The duration of the MCT intervention program was 15 weeks, and it was administered on an individual basis in semiweekly, 60-minute sessions. The first part of the MCT intervention was computer-based, and it lasted for 30 minutes. Using the commercially available RehaCom software package—a specially designed input panel that can be easily used by elderly people, accompanied by a large screen—the intervention group received cognitive training in several domains with an emphasis on episodic and delayed memory, attention, processing speed, and executive functions. All participants began the training at the beginner level of the RehaCom software. The training modules automatically adapted the training tasks to the user's level of performance. It provided the opportunity to train patients on several levels of difficulty and length of sessions, and according to whether the patient succeeded or failed the task, the difficulty levels were automatically adjusted to meet the patient's ability. At the end of the training session, the therapist could review the results from the RehaCom result screen.

Furthermore, special prominence was given to the improvement of patients' language skills, and therefore, the second part of the MCT included language exercises with pen and paper. Since there is no software package available in Greek for language skills, we collaborated with a linguist for the creation of a structured language intervention. The language intervention contained exercises of morphology, syntax, semantics, naming, verbal fluency, and word recall with a progressive increase in difficulty in each category. Patients moved at different levels and achieved mastery at their own pace, while the language therapist, throughout the whole program, was taking into consideration each patient's individual needs. Each participant was given extra cognitive and language tasks for practice at home, in a weekly basis, in order to give them the opportunity to work on their own and to get a clearer view of their skills and their difficulties. Both groups were evaluated within one week of completing the MCT.

The research protocol was approved by the Ethics Committee of the Medical School of Larissa, University of Thessaly, and it was conducted in accordance with the principles of the Declaration of Helsinki. Written consent was obtained from all the participants of the present study (or their caregivers) after having been informed of the nature of the study they would take part in.

## 3. Statistical Analysis

Baseline group characteristics are presented as mean, standard deviations, and absolute and proportion values. Normality was assessed using Kolmogorov-Smirnov graphs. Differences between the intervention and control groups at baseline assessments were estimated via the use of Pearson's chi-square and Fisher's exact test in the case of nominal variables and with Mann–Whitney *U* test in the case of scale variables. To evaluate the cognitive performance progress in each group separately over the period of 15 weeks, we compared the paired mean difference of the two assessments (baseline versus endpoint) in each group, using the paired sample *t*-test in case of normal distribution and Wilcoxon signed-rank test in case of nonnormal. The effect of the intervention was estimated by comparing the mean difference of the two assessments (baseline minus endpoint in each group) between the two groups (training group versus controls). ANOVA was applied in case of normal distribution. Otherwise, the Mann–Whitney *U* test was used. Cohen's *d* and the effect size of the intervention were calculated according to the site https://www.uccs.edu/lbecker/. The level of significance was set at 0.05 for all the analyses. All statistical calculations were performed using the SPSS for Windows (version 21) statistical software (SPSS Inc., Chicago, IL).

## 4. Results

**Figure 1:**
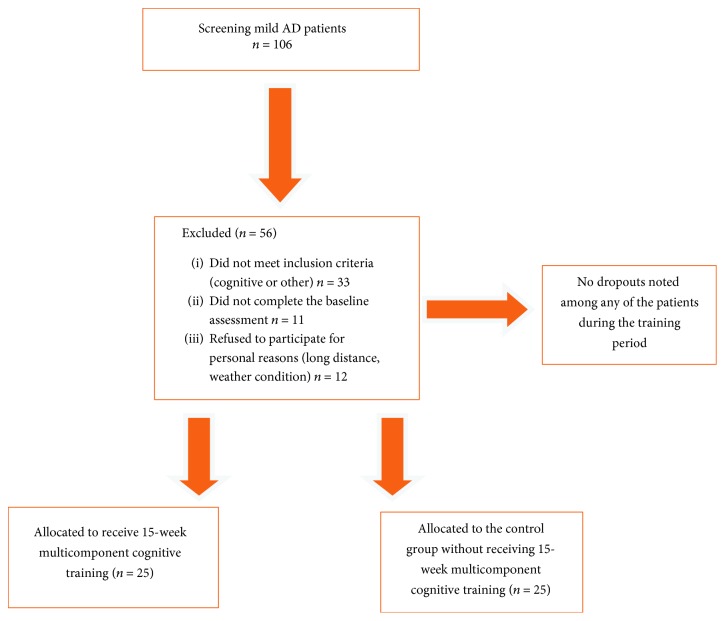
Participants' flow diagram.

From December of 2016 until July of 2017, a total of 106 mild AD patients attending the Clinical Laboratory of Speech and Language Therapy of the Technological Educational Institute of Epirus were screened for participation in the study. However, 56 patients were excluded for specific reasons (Figure 1), and therefore, 50 mild AD patients were enrolled in the study, with no dropouts noted among any of the patients during the training period. These patients were randomly assigned either to receive MCT (TG; *n* = 25) or to be placed in the control group condition (CG; *n* = 25) to receive usual standard clinical care.

The first two groups received the MCT from February 1 to April 15, 2017, and the other three groups received MCT with emphasis on linguistics from August 1 to November 15, 2017. All of the groups were then evaluated one week after the completion of the MCT with emphasis on linguistics (posttreatment).

Demographic and clinical characteristics of both groups at baseline are presented in Table 1. Baseline scores in neuropsychological tests of the training and control group are shown in Table 2. No statistically significant differences in baseline characteristics were found between the two groups.

The assessment of the paired mean difference of the two evaluations (baseline versus endpoint) in each group showed that the control group remained stable over the observation period of 15 weeks in most of the neuropsychological tests, except for delayed memory (*p* = 0.0085) and TMT A (*p* = 0.001), which deteriorated (Table 3). On the contrary, the training group improved their cognitive performance in delayed memory (*p* ≤ 0.001), recognition (*p* = 0.0284), clock-drawing test (*p* = 0.01), digit forward test (*p* ≤ 0.001), digit backward test (*p* = 0.001), TMT A (*p* ≤ 0.001), and TMT B (*p* = 0.017). Although they generally improved, the endpoint performance of the training group in word learning (*p* = 0.15) had not significantly improved (Table 3).

**Figure 2:**
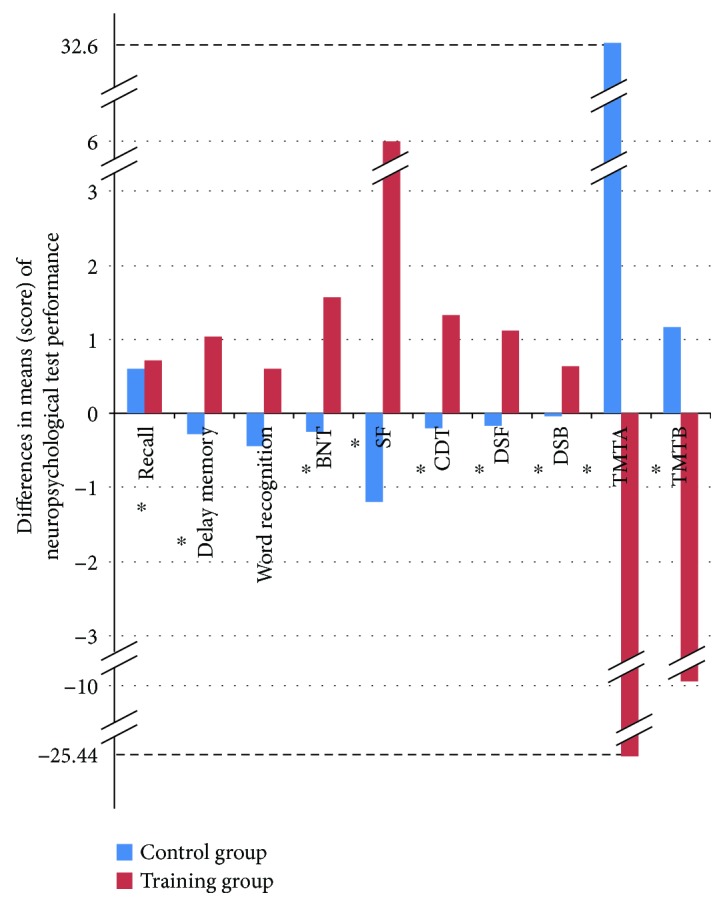
Comparison of the mean difference (score) of the two assessments (baseline minus endpoint in each group) between the two groups (training group versus controls). Recall; delayed memory; word recognition; BNT: Boston Naming Test; SF: semantic fluency; CDT: clock-drawing test; DSF: digit span forward; DSB: digit span backward; TMT A: trail-making test A; TMT B: trail-making test B. ^∗^Statistical significant values (*p* < 0.05).

Comparison between the two groups (training group versus controls) in the mean difference of the two neuropsychological assessments (baseline minus endpoint in each group) presented a significant effect of the intervention on the SF (*p* ≤ 0.001), BNT (*p* ≤ 0.001), delayed memory (*p* ≤ 0.001), word recognition (*p* = 0.008), TMT A (*p* ≤ 0.001), TMT B (*p* = 0.003), DSF (*p* ≤ 0.001), CDT (*p* ≤ 0.001), and DSB (*p* = 0.004), while no significant difference was noted for the recall of the study (Table 4). The absolute value of effect size of the training ranged from 0.02 for word recognition to 0.69 for SF. The beneficial effect of the MCT cognitive training with emphasis on linguistics in the training group when compared to the control group is depicted in Figure 2.

## 5. Discussion

The present study provides preliminary evidence that MCT improves cognition and language functions in general. Specifically, our results showed that MCT had a significant impact on delayed memory, visuospatial abilities, and executive functions. Considering the limited pharmacological therapies available for AD, concurrent computer-based MCT may represent an additional, personalized tool for the management of AD patients.

The results of the present study confirmed our first hypothesis, since all cognitive domains were improved after the computer-assisted cognitive-language-training intervention program and patients who underwent the MCT had better performance than the control group. Furthermore, our secondary hypothesis was supported. The training group had verbal positive feedback on daily activities and functional communication.

Our results are similar to those of previous studies that reported notable improvement in delayed memory [22], visuospatial abilities [21], executive function, and working memory [22] after intervention by cognitive training, focused only on memory. Other studies, though, did not find a significant improvement in naming, semantic fluency [21, 22, 40], and attention/processing speed [16]. In these studies, however, cognitive training was applied only by focusing on cognitive exercises without additional information. These findings come in contrast with our results.

Our results hint towards the considerable efficacy of MCT in naming, semantic fluency, and attention/processing speed in the training group. Moreover, a mild improvement at recall and recognition was also observed, without reaching the statistical significance threshold however. To the best of our knowledge, the effect of cognitive training in these specific domains had not been previously investigated.

AD destroys neurons and their connections in parts of the brain involved in memory. Later on, it affects cerebral areas responsible for language, reasoning, and social behavior. Eventually, many other areas of the brain are also damaged [41–43]. Since current pharmacological approaches for the cognitive decline in AD are insufficient, computer-based MCT should eventually be considered an alternative option to prevent or delay the cognitive impairment in early-stage AD patients.

To sum up, our study revealed a significant effect of MCT in almost every neuropsychological test. The existing cognitive training [16, 20–22] has a limited impact on most neuropsychological tests, probably because it does not approach cognitive and language deficits wholly. The multidomain cognitive training (MCT) offers a computer-based, personalized, and user-friendly approach for patients with AD, which simultaneously focuses on several domains.

Certain limitations of the present study need to be acknowledged. Firstly, our study had a relatively short follow-up period. Thus, a longer follow-up period would give more robust and accurate results [44]. Moreover, our study lacks analysis for additional confounders, such as the use of specific pharmacological treatments among participants, participants' APOE-*ε*4 status, along with other environmental factors that may affect the neuropsychological performance of AD patients [45, 46]. Moreover, a possible Hawthorne effect in the intervention cannot be completely excluded [47]. In addition, the lack of virtual intervention in the control group and the frequent contact of the patients of the training group with the therapist may have contributed to the positive results. Finally, considering the relatively small sample size, our study might be underpowered to examine the complete and absolute effect of MCT on AD.

## 6. Conclusions

This study showcases the beneficial impact of MCT with emphasis on linguistics and cognitive performance on patients with early-stage AD. Considering the limited efficacy of the current pharmacological therapies in AD and the limited impact of other cognitive trainings as well, computer-based MCT may represent an additional enhancing approach in early-stage AD patients. Moreover, this data is promising, in view of developing training methods to delay cognitive and language decline in early-stage AD patients. Further studies applied in larger cohorts with a priori sample size calculation are of great necessity in order to properly elucidate the effect of MCT in the progress of these patients. Emphasis should be placed on different MCI subgroups and the efficacy of multidomain cognitive MCT in linguistic deficits. If a suggestion may be done, future studies should also take aim at cognitive and language deficits when patients are diagnosed with MCI before it progresses to early-stage Alzheimer's disease, so as to properly investigate whether MCT helps in preventing AD.

## Figures and Tables

**Table 1:** Demographic and clinical characteristics of the training and control groups at baseline.

	Training group (*N* = 25)	Control group (*N* = 25)	*p* value
Gender			
Males, *N* (%)	9 (0.36)	5 (0.20)	0.345^a^
Females, *N* (%)	16 (0.64)	20 (0.80)	
Years of schooling, mean (SD)	8.08 (±3.01)	8.92 (±2.83)	0.238^b^
Age (years), mean (SD)	76.24 (±5.14)	76.32 (±5.38)	0.838^b^
GDS (score), mean (SD)	2.40 (±1.61)	3.28 (±2.30)	0.202^b^
MoCA (score), mean (SD)	16.76 (±1.33)	16.00 (±1.56)	0.108^b^
IADL (score), mean (SD)	13.60 (±2.10)	12.64 (±1.57)	0.102^b^

GDS: Geriatric Depression Scale; MoCA: Montreal Cognitive Assessment; IADL: Instrumental Activities of Daily Living; ^a^chi-square test; ^b^Mann–Whitney *U* test.

**Table 2:** Neuropsychological test scores of the training and control groups at baseline.

	Training group (*N* = 25)	Control group (*N* = 25)	*p* value
Recall, mean (SD)	17.44 (±3.11)	16.60 (±3.26)	0.356^a^
Delayed memory, mean (SD)	0.16 (±0.37)	0.40 (±0.50)	0.061^b^
Word recognition, mean (SD)	18.08 (±0.26)	18.40 (±0.25)	0.414^b^
BNT, mean (SD)	11.84 (±1.57)	11.64 (±1.52)	0.572^b^
SF, mean (SD)	22.12 (±6.51)	23.36 (±7.44)	0.534^a^
CDT, mean (SD)	8.96 (±2.23)	9.72 (±1.93)	0.401^b^
DSF, mean (SD)	5.48 (±0.71)	5.04 (±0.94)	0.085^b^
DSΒ, mean (SD)	3.68 (±0.75)	3.36 (±0.81)	0.098^b^
TMT A, mean (SD)	177.24 (±45.88)	177.56 (±56.02)	0.982^a^
TMT B, mean (SD)	300.00 (±0.00)	297.84 (±10.80)	0.317^b^

BNT: Boston Naming Test; SF: semantic fluency; CDT: clock-drawing test; DSF: digit span forward; DSΒ: digit span backward; TMT A: trail-making test A; TMT B: trail-making test B; ^a^ANOVA; ^b^Mann–Whitney *U* test.

**Table 3:** Mean score, standard deviation, *p* value, and pre- and postassessment in the control and training groups.

	Control group			Training group		
	Preassessment	Postassessment	*p* value	Preassessment	Postassessment	*p* value
	Mean (SD)	Mean (SD)		Mean (SD)	Mean (SD)	
Recall	16.60 (3.26)	16.20 (2.45)	0.33^b^	17.44 (3.66)	18.16 (3.48)	0.151^b^
Delayed memory	0.40 (0.50)	0.12 (0.33)	**0.08 ** ^b^	0.16 (0.37)	1.20 (1.08)	**≤0.001 ** ^b^
Word recognition	18.40 (1.25)	17.96 (1.48)	0.20^b^	18.08 (1.32)	18.68 (1.28)	0.028^b^
BNT	11.64 (1.32)	11.40 (1.30)	0.22^b^	11.84 (1.57)	13.40 (1.04)	**≤0.001 ** ^b^
SF	23.36 (7.44)	22.16 (6.31)	0.13^b^	22.12 (6.05)	28.16 (6.08)	**≤0.001 ** ^a^
CDT	9.72 (1.93)	9.52 (1.36)	0.24^b^	8.96 (2.22)	10.28 (2.59)	**0.01 ** ^a^
DSF	5.04 (0.93)	4.88 (1.13)	0.35^b^	5.48 (0.71)	6.60 (1.35)	≤0.001^b^
DSB	3.36 (0.81)	3.32 (0.98)	0.80^b^	3.68 (0.75)	4.32 (0.75)	0.001^b^
TMT A	177.56 (56.02)	210.16 (66.58)	**0.01 ** ^b^	177.24 (45.88)	151.80 (39.48)	≤0.001^b^
TMT B	297.84 (10.80)	299.00 (5.00)	0.32^b^	300 (00.00)	290.60 (24.67)	0.017^b^

BNT: Boston Naming Test; SF: semantic fluency; CDT: clock-drawing test; DSF: digit span forward; DSΒ: digit span backward; TMT A: trail-making test A; TMT B: trail-making test B; ^a^paired sample *t*-test; ^b^Wilcoxon signed-rank test.

**Table 4:** The effect of the intervention (estimated by comparing the mean difference of the two neuropsychological assessments (baseline minus endpoint in each group) between the two groups (training group versus controls)) in the training group compared to the control group.

	Training group (*N* = 25)	Control group (*N* = 25)	*p* value	Cohen's *d*	Effect size
Recall, mean difference (95% CI)	0.72 (−0.73, 2.17)	0.60 (−0.36, 1.56)	0.887^a^	0.04	0.02
Delayed memory, mean difference (95% CI)	1.04 (0.60, 1.48)	−0.28 (−0.47, −0.09)	≤0.001^b^	1.12	0.48
Word recognition, mean difference (95% CI)	0.60 (0.02, 1.18)	−0.44 (−1.11, 0.23)	0.008^b^	0.68	0.32
BNT, mean difference (95% CI)	1.56 (1.11, 2.01)	−0.24 (−0.62, 0.14)	≤0.001^b^	1.79	0.67
SF, mean difference (95% CI)	6.04 (4.33, 7.75)	−1.20 (−2.62, 0.22)	≤0.001^a^	1.89	0.69
CDT, mean difference (95% CI)	1.32 (0.64, 2.00)	−0.20 (−0.54, 0.14)	≤0.001^b^	1.16	0.50
DSF, mean difference (95% CI)	1.12 (0.60, 1.64)	−0.16 (−0.60, 0.28)	≤0.001^b^	1.09	0.48
DSB, mean difference (95% CI)	0.64 (0.33, 0.95)	−0.04 (−0.37, 0.29)	0.004^b^	0.88	0.40
TMT A, mean difference (95% CI)	−25.44 (−34.99, −15.89)	32.60 (11.11, 54.09)	≤0.001^a^	−1.44	−0.58
TMT B, mean difference (95% CI)	−9.40 (−19.59, 0.79)	1.16 (−1.23, 3.55)	0.003^b^	−0.59	−0.28

BNT: Boston Naming Test; SF: semantic fluency; CDT: clock-drawing test; DSF: digit span forward; DSΒ: digit span backward; TMT A: trail-making test A; TMT B: trail-making test B; ^a^ANOVA; ^b^Mann–Whitney *U* test.

## Data Availability

The data used to support the findings of this study are available from the corresponding author upon request.
